# Mogamulizumab-induced interface dermatitis drug rash treated successfully with methotrexate and extracorporeal photopheresis in a patient with Sézary syndrome

**DOI:** 10.1016/j.jdcr.2020.12.035

**Published:** 2021-01-12

**Authors:** Ilana D. Breen, Caitlin M. Brumfiel, Meera H. Patel, Allison C. Rosenthal, William G. Rule, David J. DiCaudo, Fiona E. Craig, Mark R. Pittelkow, Aaron R. Mangold

**Affiliations:** a Mayo Clinic Alix School of Medicine, Scottsdale, Arizona; b Mayo Clinic Arizona, Dermatology, Scottsdale, Arizona; c Mayo Clinic Arizona, Hematology Oncology, Phoenix, Arizona; d Mayo Clinic Arizona, Radiation Oncology, Phoenix, Arizona; e Mayo Clinic Arizona, Laboratory Medicine and Pathology, Phoenix, Arizona

**Keywords:** Cutaneous T-cell lymphoma, extracorporeal photopheresis, interface dermatitis, methotrexate, mogamulizumab, Sézary, CCR4, C-C chemokine receptor 4, ECP, extracorporeal photopheresis, MF, mycosis fungoides, SS, Sézary syndrome

## Introduction

Sézary syndrome (SS) is defined by erythroderma, the presence of circulating malignant T cells in the peripheral blood, and generalized lymphadenopathy. SS is associated with a low or high disease burden depending on the presence of less or greater than 5000/μL circulating malignant cells.^1^ High-risk disease requires more aggressive treatment with a combination of extracorporeal photopheresis (ECP), interferon, or bexarotene, or monotherapy with romidepsin or mogamulizumab.

Mogamulizumab was approved by the U.S. Food and Drug Administration for relapsed or refractory mycosis fungoides (MF) or SS following failure of at least one previous course of systemic therapy.^2^ Mogamulizumab is a monoclonal antibody directed toward the C-C chemokine receptor 4 (CCR4), which is expressed in T-cell malignancies including MF and SS, and was found to be highly effective in the blood compartment with a 68% response and in SS with a 37% overall response rate.^3^ In the phase III MF/SS clinical trial, grade 2 and 3 drug rashes were seen in approximately 1 in 4 patients on mogamulizumab.^3^ Several post-marketing surveillance case studies of mogamulizumab for the treatment of adult T-cell leukemia/lymphoma reported Stevens-Johnson syndrome/toxic epidermal necrolysis, underscoring the importance of swift recognition and effective treatment induction.^4^

Herein, we present a case of high-burden SS with an atypical and corticosteroid-refractory drug rash while on mogamulizumab, treated successfully with ECP and methotrexate.

## Case report

A 56-year-old man presented with a two-year history of progressive erythema and pruritus. Physical examination demonstrated erythroderma with adenopathy. Positron emission tomography/computed tomography scan was unremarkable. A punch biopsy revealed atypical lymphoid infiltrate, and flow cytometry revealed lymphocytosis with 93% CD4+/CD26-clonal T cells, consistent with a diagnosis of SS with high disease burden, given the absolute lymphocyte count of 11,865/μL. Initial therapy with romidepsin failed, and the patient was subsequently transitioned to mogamulizumab. Following 5 cycles (1 mg/kg on days 1,8,15, and 22 of a 28-day cycle of cycle 1 and then 1 mg/kg on days 1 and 15 in cycles 2 and beyond, every 28 days), he achieved a complete blood response with no evidence of phenotypically aberrant T cell population as noted on peripheral blood flow cytometry.

**Fig 1:**
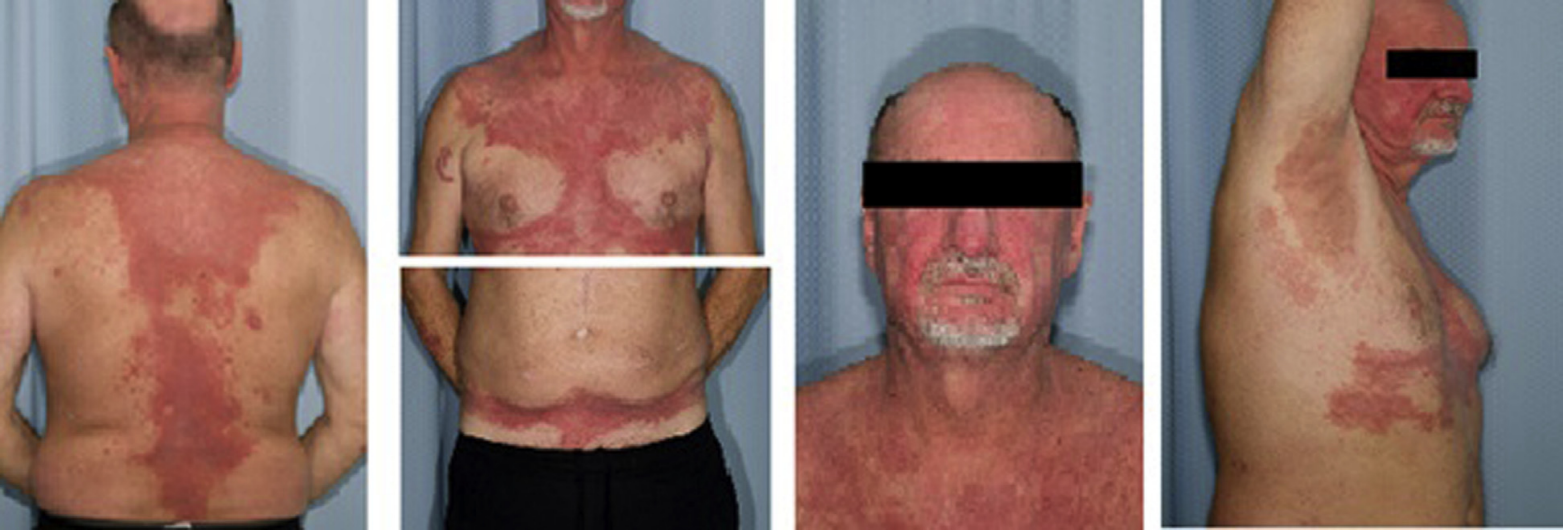
Dermatitic rash of the head and neck and atypical psoriasiform plaques and patches concentrated on the chest, back, forearms, and flexural crease of the abdomen.

**Fig 2:**
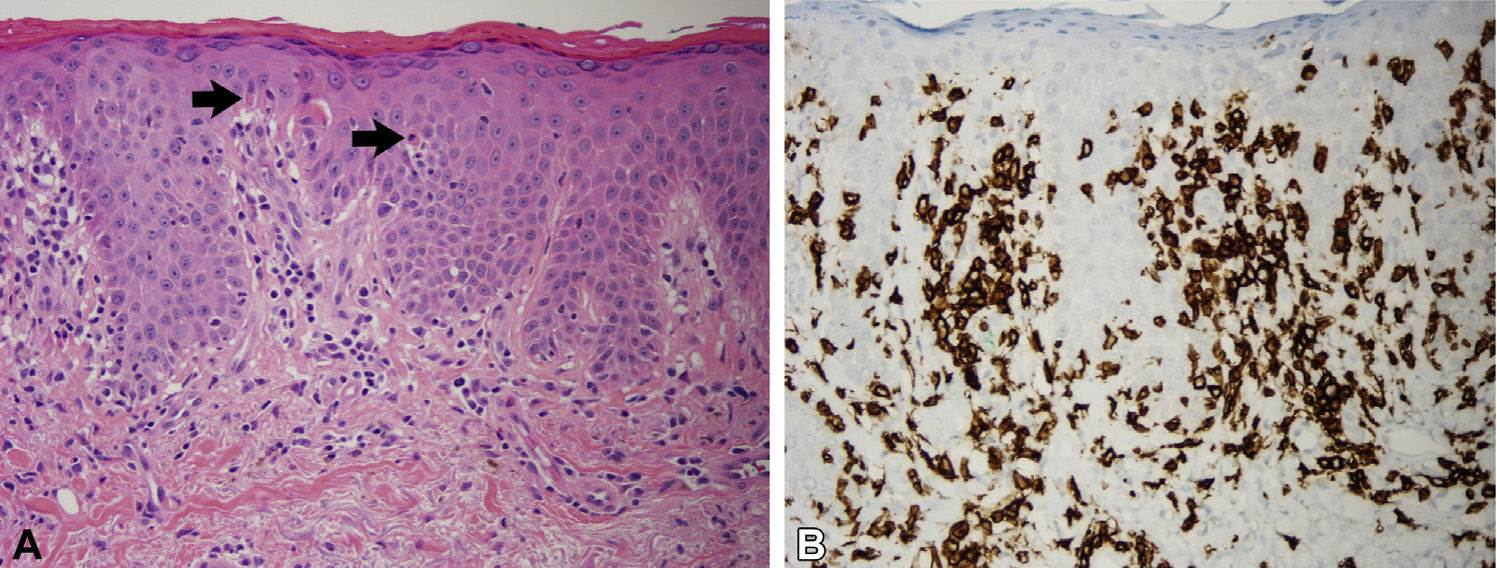
Histopathology of left upper chest punch biopsy. **A**, Mild lymphocytic infiltrate along the dermal-epidermal junction with scattered apoptotic keratinocytes (arrows) within the epidermis (Hematoxylin-eosin stain; original magnification: ×200.) **B**, CD8+ T cells extending into the epidermis (CD8 staining, original magnification: ×200.)

**Fig 3:**
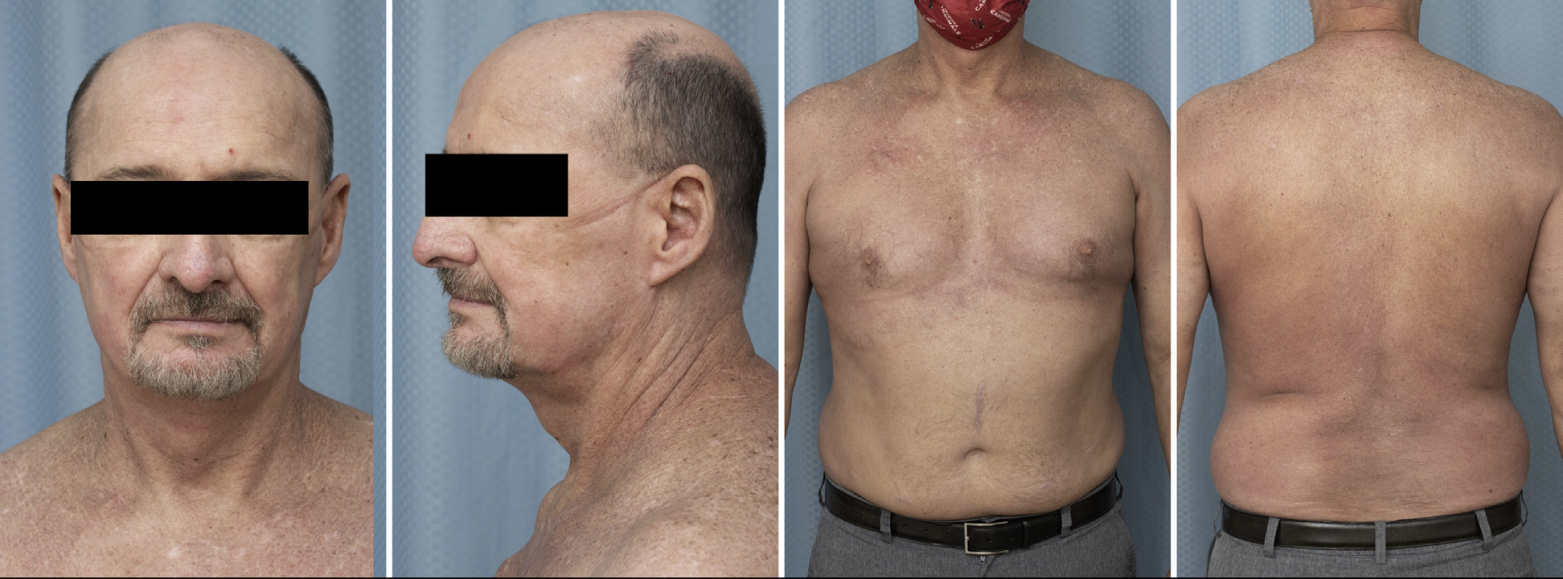
Images of the patient's drug rash following treatment with methotrexate and extracorporeal photopheresis.

However, several weeks after a course of cefadroxil for *Staphylococcus* cellulitis, he developed angioedema and a progressive, pruritic grade 3 rash over the chest, axilla, and groin (Fig 1). Mogamulizumab was discontinued. The angioedema occurred in a single episode, and workup for acquired and hereditary angioedema was negative. Differential diagnosis at the time included progressive SS, symmetrical drug-related intertriginous and flexural exanthema, pityriasis rubra pilaris, and drug-provoked autoimmune disorders (e.g. dermatomyositis—Wong's type). Extensive workup including antinuclear antibodies, extractable nuclear antigens, and myomarker and necrotizing myopathy panels, was negative, ruling out a drug-provoked autoimmune reaction. Punch biopsy of the left upper chest revealed interface dermatitis with a CD8 predominant phenotype, ruling out pityriasis rubra pilaris-like drug reaction and symmetrical drug-related intertriginous and flexural exanthema, favoring the diagnosis of cell-poor lichenoid drug reaction, the second most common mogamulizumab-associated rash reaction pattern^5^ (Fig 2). Notably, the CD8+ immunophenotype of the cells in the drug eruption was different from that of the CD4+ immunophenotype of the patient's SS. The rash exhibited an inadequate, partial response to oral prednisone 40 mg daily for 3 months. Ultimately, the patient was tapered from steroids and treated with ECP every 2 weeks for 5 months in total, with the addition of methotrexate 25 mg weekly initiated 2 months into ECP. This regimen led to near total resolution of his rash (Fig 3).

## Discussion

Despite the high frequency of mogamulizumab-associated drug rashes, there is a dearth of literature describing treatment strategies for steroid-refractory cases. The mechanism of action of mogamulizumab accounts for the development of lichenoid drug rashes. Mogamulizumab targets the CCR4 receptor expressed on peripherally circulating diseased T cells; but, it also modulates receptors on regulatory T cells and leads to their depletion, which can cause immune activation.^2^ A recent study reported that development of chronic granulomatous drug eruption during mogamulizumab therapy may signify a durable response to the drug.^6^ This correlation is attributed to the anti-CCR4 effects of mogamulizumab, which lead to the T helper 1 polarization and regulatory T cell suppression mechanistic actions responsible for treating cutaneous T-cell lymphoma.^6^ Similarly, our patient's cell-poor lichenoid drug rash on mogamulizumab coincided with remission.

The recruitment and proliferation of cytotoxic T cells observed in lichenoid drug reactions closely resemble those of graft-versus-host-disease. Steroid-refractory graft-versus-host-disease can be treated with ECP, methotrexate, or a combination of these.^7^^,^^8^ Methotrexate is particularly well suited for both SS and lichenoid reactions, as it is known to suppress dysregulated T cells with toxicity, specifically toward highly proliferative lymphocytes. ECP leads to anti-inflammatory cytokines and stimulation of regulatory T cells, which replenishes regulatory T-cell responses and confers the immunomodulatory effects responsible for counteracting alloimmunity in graft-versus-host-disease and autoimmunity in lichenoid drug reaction.^9^ Additionally, several reported cases of SS have been treated successfully with the combination of methotrexate and ECP.^10^ The rescue of regulatory T-cell functionality by ECP may lead to improved tolerability of mogamulizumab, when the 2 agents are used in combination for treatment of MF/SS and requires further study.

## Conflicts of interest

Dr. Mangold currently provides scientific advisory for Eli Lilly and Kirin. Dr. Mangold is a clinical investigator at Eli Lilly, Novartis, Sun Pharmaceutical, Pfizer, Acetilion, Incyte, Corbus, MiRagen, Solagenix, and Regeneron. The rest of the authors have no conflicts to disclose.
